# Managing Postangiography Radial Artery Pseudoaneurysms: Systematic Review of the Published Literature

**DOI:** 10.1016/j.jscai.2026.104263

**Published:** 2026-02-24

**Authors:** Haidar Hajeh, Aditya Desai, Amer Muhyieddeen, Prabhdeep Sethi, Yasin Hussain, Tanawan Riangwiwat

**Affiliations:** a Cardiology Department, School of Medicine, University of California Riverside, Riverside, California; b Cardiology Department, Rush University Medical Center, Chicago, Illinois

**Keywords:** pseudoaneurysm, radial artery, vascular access, vascular complications

## Abstract

**Background:**

With the growing use of radial access in coronary angiography, rare but significant complications such as radial artery pseudoaneurysms are increasingly encountered. This arises when arterial wall disruption leads to a soft tissue hematoma with persistent communication with the arterial lumen. Literature on management remains scarce and consists of a few case reports.

**Methods:**

We conducted a systematic review of cases describing radial artery pseudoaneurysms following coronary angiography from January 1992 to February 2025 using PubMed, Scopus, and Google Scholar. Data extracted included patient demographics, anticoagulant use, clinical presentation, pseudoaneurysm dimensions, timing of symptom onset, and treatment strategies with associated outcomes.

**Results:**

A total of 56 publications comprising 75 patients were analyzed. Mean age was 70.9 years, with predominance in women (58.3%), albeit statistically nonsignificant (*P* = .157). All patients had access site swelling, and half had pain. Most pseudoaneurysms developed within the first month postprocedure (81.6%). Chronic anticoagulant use was reported in 58.3% of patients, with warfarin being the most common agent. Four primary treatment modalities were reported: compression (n = 24), thrombin injection (n = 9), percutaneous interventions (n = 6), and surgery (n = 36). Compression was the most frequently used but least effective method, with a 58.5% success rate as compared to 100% in the other methods.

**Conclusions:**

Radial artery pseudoaneurysms are rare but potentially serious complications of transradial coronary procedures. Although compression is commonly attempted, invasive treatments offer superior efficacy.

## Introduction

Coronary angiography has been the mainstay for diagnosing coronary arterial disease since the 1960s. In the later years, there has been a shift toward using the radial artery as a default access site in place of the femoral artery because of the lower risk of serious access site complications and improved patient comfort. It, however, introduces a new set of complications, including radial artery pseudoaneurysms. This happens when the arteriotomy site allows blood to escape into the surrounding soft tissue, forming a hematoma while maintaining flow from the artery lumen to the hematoma and backward. Radial artery pseudoaneurysms are a rare complication of radial access, with a reported incidence of 0.09% in a large paper including 10,676 post–coronary angiography patients.^1^ It presents as swelling at the access site, and it is frequently associated with pain, which makes patients seek medical attention. Some papers have reported spontaneous resolution of radial artery pseudoaneurysms; however, most cases require intervention. We must acknowledge the possible reporting bias where many cases of spontaneous resolution may not be reported, as patients may not seek medical attention. Given their rarity, there is very little literature published describing their management, which mainly consists of individual case reports and short review articles.^2^ This leaves cardiologists with very little guidance on how to manage this complication.

## Methods

We performed a systematic review of the available literature on PubMed, Scopus, and Google Scholar in the period from January 1992 until February 2025. Study design was developed in accordance with the Preferred Reporting Items for Systematic Reviews and Meta-Analyses (PRISMA) method. We looked for case reports or case series that described patients who developed radial artery pseudoaneurysms after a coronary catheterization procedure to answer the questions “What are the characteristics of patients who develop radial artery pseudoaneurysms after coronary angiography,” “Is there an association between anticoagulant use and this complication,” and “What are the management methods used to treat this complication and how effective are they.” We searched the databases using variations of “radial artery pseudoaneurysm” as keywords for the pathology, and variations of “coronary angiography” or “left heart catheterization” as keywords for the procedure. We included a total of 551 publications: 160 articles from PubMed, 191 articles from Scopus, and 200 articles from Google Scholar in the screening.

We excluded duplicates, publications that were not case reports/case series, papers with missing text, cases describing procedures that were not coronary angiography or percutaneous coronary interventions, cases using distal radial artery access, or cases with concomitant complications that would affect the management decision (eg, arteriovenous fistula formation). Of note, we included publications other than case reports/case series only if they described individual patients of the studied population.

**Figure 1:**
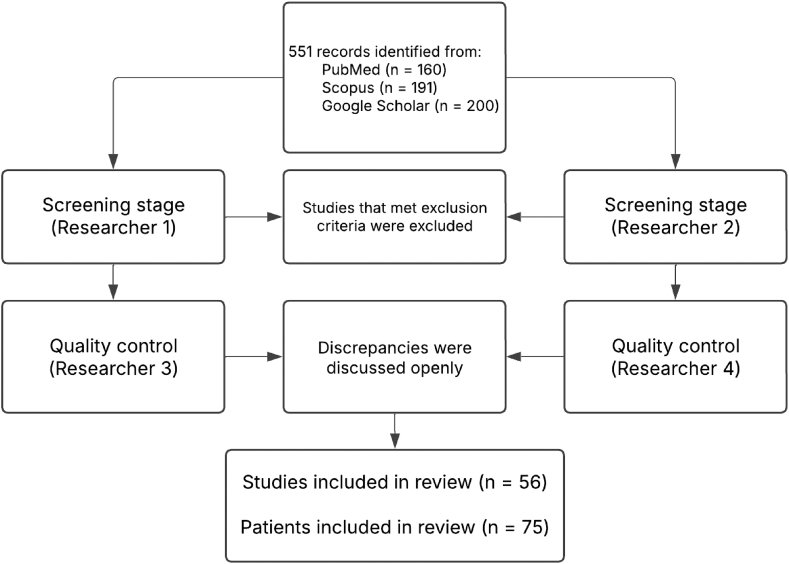
**Method of literature search.** A total of 56 publications describing 75 patients were included in the final analysis.

This process was done twice by 2 researchers (H.H., A.D.) independently. Results from each group underwent a quality control check by 2 researchers (A.M., P.S.) who excluded publications that still met the exclusion criteria from each of the groups. Discrepancies between the 2 groups at the end were settled openly between all 4 researchers (Figure 1).

Data extraction from the final 56 publications was performed by the first author. Results were then checked by the second author.

Data collected included age, sex, symptoms, time of onset of symptoms from time of procedure, pseudoaneurysm dimensions on ultrasonography, chronic use of anticoagulants, and failed/successful methods used in managing the pseudoaneurysm. The efficacy of each intervention was evaluated using the ratio of cases successfully controlled with an intervention to the total number of cases that used the intervention. Collected data were analyzed using the χ^2^ test for categorical data and presented as counts, averages, and percentages with the corresponding *P* values.

Of note, institutional review board approval and individual patient consent were not required for this article, given the “literature review” nature of this paper and given that it does not involve human subject data other than what is already published in the literature.

## Results

The final analysis (Supplemental Table S1) included 56 publications from 24 countries and consisted of 1 retrospective study, 6 case series, and 49 case reports.^3^, ^4^, ^5^, ^6^, ^7^, ^8^, ^9^, ^10^, ^11^, ^12^, ^13^, ^14^, ^15^, ^16^, ^17^, ^18^, ^19^, ^20^, ^21^, ^22^, ^23^, ^24^, ^25^, ^26^, ^27^, ^28^, ^29^, ^30^, ^31^, ^32^, ^33^, ^34^, ^35^, ^36^, ^37^, ^38^, ^39^, ^40^, ^41^, ^42^, ^43^, ^44^, ^45^, ^46^, ^47^, ^48^, ^49^, ^50^, ^51^, ^52^, ^53^, ^54^, ^55^, ^56^, ^57^, ^58^

The final analysis included 75 patients (Table 1) composed of 30 men and 42 women with a mean age of 71 years. Sex distribution showed a predominance in women (58.3%). It was, however, not statistically significant (*P* = .157). Sex was reported as included in the original papers, and the choice of terminology “male” vs “female” was at the discretion of the authors of the individual publication.

**Table 1:** Results.

Characteristic	N = 75	*P* value
Age, y	70.9 ± 12.6 (n = 72)	–
Sex		.157
Male	30/72 (41.7)	
Female	42/72 (58.3)	
Symptoms		–
Swelling	68/68 (100)	–
Pain	35/68 (51.4)	–
Bleeding	3/68 (4.4)	–
Neurological symptoms	2/68 (2.9)	–
Ulceration	1/68 (1.4)	–
Onset of symptoms, d	25.2 (n = 71)	–
Onset of symptoms after procedure		
Within 1 week	41 (57.7)	–
Within 1 month	58 (81.6)	–
Within 3 months	66 (92.9)	–
Longest diameter, mm	26.2 (n = 42)	–
Chronic anticoagulant use		.196
Yes	35/60 (58.3)	
No	25/60 (41.7)	
Type of anticoagulant		
Warfarin	18/34 (52.9)	.013
Enoxaparin	4/34 (11.7)	.013
Apixaban	8/34 (23.5)	–
Rivaroxaban	2/34 (5.8)	–
Unspecified DOAC	2/34 (5.8)	–
Comulative DOAC	12/34 (35.2)	.013
Successful management		
Compression	24/75 (32)	–
Percutaneous	6/75 (8)	–
Thrombin injection	9/75 (12)	–
Surgery	36/75 (48)	–

Values are mean ± SD or n (%).

DOAC, direct oral anticoagulant.

All patients presented with wrist swelling at the arteriotomy site, with pain being the second most commonly reported symptom occurring in 51.4% of the patients. Bleeding, neurological dysfunction, and ulceration were present less frequently (4.4%, 2.9%, and 1.4% of the total patients, respectively). None of the included publications reported compartment syndrome or hand ischemia; however, 2 publications reported paresthesia in the fingers with mild thumb abduction weakness in one of them. Paresthesia resolved and muscle weakness improved significantly, albeit not resolved, after the resolution of the pseudoaneurysm.

The onset of symptoms had a median of 7 days after the index procedure. It is noteworthy that the onset of symptoms varied significantly among reported cases, with the earliest occurring on the same day as the index procedure and the latest presenting up to 1 year later. Further analysis showed that 7 patients (9.9%) developed radial artery pseudoaneurysm on the same day as the index procedure, 10 patients (14.1%) developed it on the following day, and 41 patients (57.7%) developed it within the first week. This illustrates the importance of examining the access site within 1 week following the procedure.

Many cases reported the dimensions of the pseudoaneurysm with a reported mean of the longest diameter of 26.2 ± 15.1 mm. The neck length was not consistently reported, although it played an important role in deciding suitability for thrombin injection, as detailed in the Discussion section.

It is important to note that more than half the patients (58.3%) were on chronic anticoagulants. Among those patients, the majority were being treated with warfarin (52.9% with *P* = .013).

There were 4 reported methods for managing radial artery pseudoaneurysms, which included compression, thrombin injection, percutaneous interventions, and surgery.

Compression was accomplished using compression devices (eg, radial inflatable compression bands), compressive bandages, or ultrasound (US)-guided compression. It aimed to interrupt the flow between the artery and the pseudoaneurysm, allowing for the body to reabsorb the residual hematoma.

Compression techniques varied between the different publications. The compression device (inflatable radial compression band) was successful in resolving the pseudoaneurysm in 66.7% of the times it was used and had the highest success rate. US-guided compression was successful in 44.4% of the cases, and manual/bandage compression was the least successful, with a success rate of 30.8% of the cases in which it was used.

Thrombin injection was performed in 9 cases, with curative results in all cases.

Percutaneous interventions (6 cases) included isolating the radial artery from the pseudoaneurysm using a covered stent (4 cases), placing a long sheath via a distal radial arterial access that covers the arteriotomy (1 case), or placement of endovascular coils (1 case). Surgical interventions included resection of the affected radial artery segment and arteriorrhaphy.

It is noteworthy that all methods were 100% curative except compression, which was effective in 58.5% of the cases (Central Illustration). Resolution of the pseudoaneurysm was defined as resolution of the pseudoaneurysm symptoms and a benign physical examination on follow-up with or without ultrasonographic confirmation. Table 2 shows the efficacy of management methods presented as the number of times the pseudoaneurysm was successfully managed with a method over the total number of times that method was used (Table 2).

**Central Illustration:**
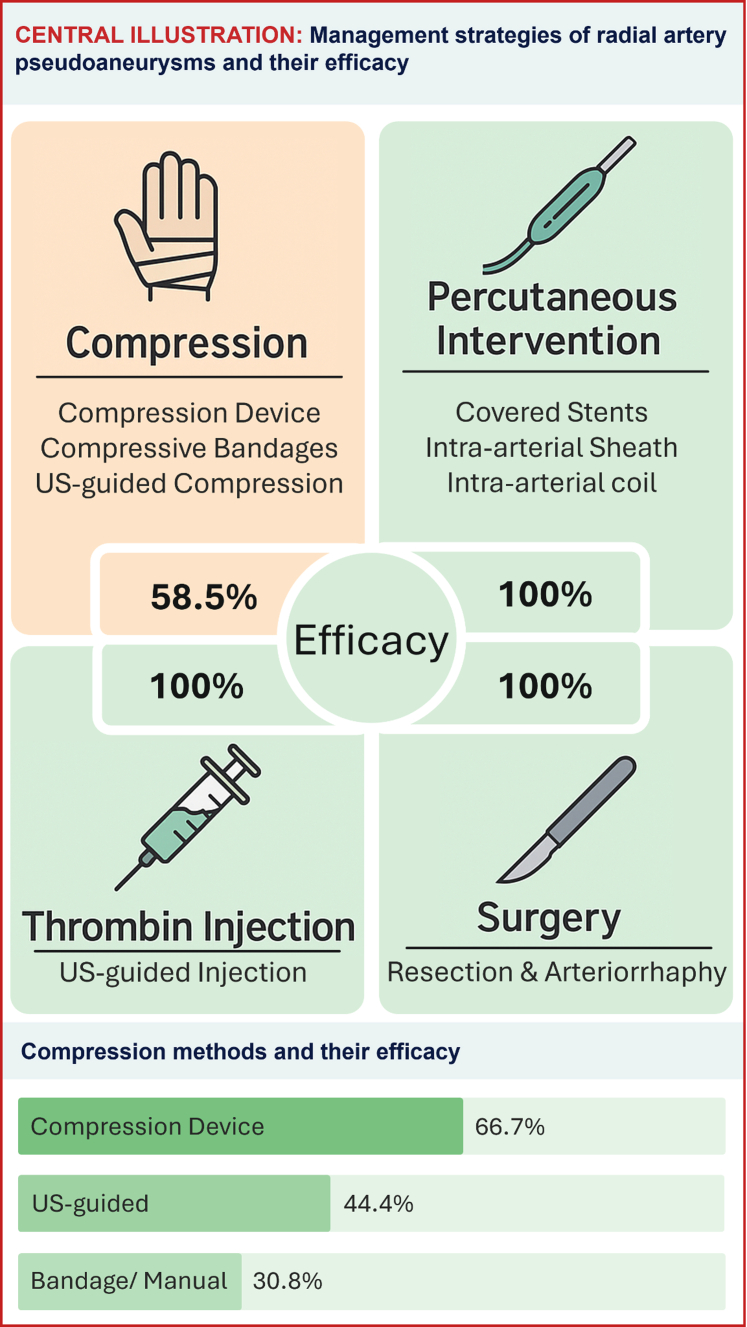
**Management strategies of radial artery pseudoaneurysms (including the specific compression methods) and their efficacy.** US, ultrasound.

**Table 2:** Efficacy of management methods.

Method	Efficacy
Compression	58.5% (24/41)
Percutaneous intervention	100% (6/6)
Thrombin injection	100% (9/9)
Surgery	100% (36/36)

Values are % (n/N).

## Discussion

Patients who developed radial artery pseudoaneurysm had a mean age of 71 years with 58% predominance in women. It is important, however, to consider the context of this sex distribution, where coronary angiography is performed in men more than in women in most cardiovascular publications.^59^ Having predominance in women in the occurrence of pseudoaneurysms in the cohort of patients undergoing coronary angiography, in which the majority of these patients are men, points to a possible higher probability of developing this complication in women than in men. Large prospective or retrospective cohorts of this complication are needed to prove this theory.

The majority of cases occurred within the first week after angiography, with a decrease in incidence over time. Examining the access site should be performed especially within the first week after the procedure, as the majority of pseudoaneurysms present during the first week. The occurrence of this complication decreases over time after that, despite reports of developing this complication up to 1 year after the index procedure.

Multiple publications have suggested a correlation between anticoagulant use and pseudoaneurysm formation. A previous study has identified anticoagulant use as a risk factor for the development of femoral artery pseudoaneurysms as well as a predictor of failure of conservative management, often necessitating invasive treatment.^59^ The findings of our paper showed similar results in the context of the radial artery, where 58.3% of patients were receiving chronic anticoagulants with a *P* value (*P* = .196). The *P* value indicates that the difference observed in this distribution can be plausibly explained by the chance of random sampling variation and that the use of anticoagulants is as prevalent as no anticoagulant use in our studied population. We question, however, if the results would remain significant when compared to the general population of patients undergoing coronary angiography, where the percentage of patients taking anticoagulants is lower than those who are not, leading to an unequal distribution. If our results of 58.3% of patients using anticoagulants were compared against the general angiography population, where the proportion is the opposite, the results may be different.

Anticoagulant use in our paper was defined as using anticoagulants chronically after the index procedure. Indications for use varied between atrial fibrillation, valve replacement, and other indications. Papers on post–coronary angiography femoral artery pseudoaneurysms showed a percentage of spontaneous resolution of the pseudoaneurysms. This happens because of the activation of the coagulation cascade in the pseudoaneurysm, leading to the interruption of blood flow from and to the pseudoaneurysm. Anticoagulation likely prevents this mechanism, leading to the persistence of the pseudoaneurysm. Several other independent risk factors have been described as well, including hypertension, diabetes, coronary artery disease, larger catheter diameter, and higher patient body mass index.^60^ These risk factors are likely similar between femoral and radial artery pseudoaneurysms; however, more data are needed to establish this relationship.

Interestingly, there was a higher proportion of warfarin use as compared to other anticoagulants, with a *P* value of .013, despite the significant decrease in warfarin use for anticoagulation in the general population after the introduction of direct oral anticoagulants (DOAC) in the absence of specific indications. This could be due to the unpredictable effects of warfarin compared to DOAC, whereas stopping DOAC for about 48 hours prior to coronary angiography is generally safe with a predicted cessation of their anticoagulant effect. This may not be true, however, in patients on warfarin, where the optimal duration of stopping the medication to achieve the subtherapeutic international normalized ratio (INR) goal is not known and differs depending on the therapeutic INR goal, metabolism, other ingested medications, and warfarin dose. It is also important to consider the possibility of a reporting bias, as most patients taking warfarin have specific indications for this medication (mechanical valve replacement, left ventricular thrombus, etc) that require close and frequent monitoring, which leads to the detection of radial artery pseudoaneurysms during one of the follow-ups.

Management of pseudoaneurysms varied widely among the reported publications. Guidelines and cardiology societies provide little guidance to operators on managing this complication, given the lack of trials and head-to-head comparisons between the different management methods.

Compression was the most widely used method because of its noninvasive nature and ease of application. However, it was the least effective. If compression is to be applied, we recommend US guidance to define the arteriotomy site where pressure should be applied in an effort to interrupt the to-and-fro flow through the arteriotomy. Compression devices, like radial inflatable compression bands, allow localizing the pressure to the radial site as compared to compressive bandages that are associated with wider pressure application and possible vein compression leading to hand edema. Patent hemostasis techniques should be used to avoid radial artery occlusion, where the pressure applied should be just enough to prevent the flow through the arteriotomy, without being high enough to occlude the artery.^61^ It is reasonable to attempt compression until a more definitive treatment option is planned. Patients should be educated in advance on the high failure rates and the likely need for a more invasive method in the majority of cases.

Thrombin injection is a minimally invasive procedure with high success rates (achieved in all 9 cases). All patients treated with thrombin injection in our paper had resolution of pseudoaneurysms. The availability of this method is highly dependent on available operator expertise. Pseudoaneurysm neck length was reported in 19 cases with an average of 2.6 mm, and it played an important role in deciding the suitability for injection. This is probably an extrapolation of previous publications that suggested that shorter necks were associated with failure of femoral artery pseudoaneurysm management with thrombin injection.^62^ Thrombin injection poses a theoretical risk of thrombin embolization into the systemic circulation and possibly causing finger ischemia. None of the cases that used thrombin injection in our paper reported this complication. A recent publication of 34 patients with radial artery pseudoaneurysms with neck length >3 mm and arteriotomy diameter <5 mm treated with thrombin injection reported an 82% success rate on the first injection attempt. Repeat injections slightly increased the success rate. It is important to note that only 64.7% of these patients had pseudoaneurysms as a result of coronary angiography, while the others had other etiologies.^63^ Our paper reported a 100% success rate of using thrombin injection compared to the 82% success rate reported by this publication. This may be due to the inclusion of radial pseudoaneurysms of different etiologies other than coronary angiography access, which may be associated with a lower success rate. Periprocedural arterial thrombus formation was noted in 5 patients in this article, giving a complication rate of 14.7% with 1 patient reporting transient ischemic symptoms in the fingers. The success rate of this method and the avoidance of complications like radial artery thrombosis are highly dependent on patient selection based on the pseudoaneurysm anatomy. Factors favoring this method include a longer neck (preferably >3 mm), smaller arteriotomy diameter (preferably <5 mm), superficial pseudoaneurysms with no vital structures in the vicinity, in addition to the availability of experienced proceduralists who are comfortable with performing such injections.

Most cases treated percutaneously included using a covered stent to cover the arteriotomy site. Delivering the stent was performed through a distal radial, ulnar, or brachial access. This allows for performing a diagnostic angiogram prior to and after deployment to prove the isolation of the artery from the pseudoaneurysm when no contrast reaches the pseudoaneurysm on postdeployment angiography. Choosing the access site for this procedure depends on the location of the pseudoaneurysm and the experience of the operator. In one case, a radial sheath was used to cover the arteriotomy site. An 8F sheath was inserted via distal radial access after assessing the radial artery diameter ultrasonographically to ensure the use of an appropriate sheath size. The sheath was used to cover the arteriotomy site and was left in place for 24 hours with a compression inflatable radial band over the site to ensure internal and external compression of the artery. Continuous saline infusion was used to maintain sheath patency. No radial artery occlusion was reported at the end of the treatment and monitoring period.^39^

Surgical intervention was used as primary therapy in 26 cases (34.7%) and as a bailout strategy after failure of primary therapy in 10 cases (13.3%). Surgical strategy consisted of resection of the defective section of the artery with arteriorrhaphy.

Multidisciplinary discussion between the patient, interventional cardiology, interventional radiology, and vascular surgery should be done to select the most suitable strategy that poses the lowest risk. A summary of the strengths and limitations of each method, in addition to a guide on when to use each method, is included in Table 3.

**Table 3:** Summary of methods to manage postangiography radial artery pseudoaneurysms.

Method	Strengths	Limitations	When to use	Complications
Compression - Device - Manual - US-guided	- Only noninvasive method	- High failure rates - Uncomfortable - Requires prolonged applications	- If refused/unsuitable for invasive methods - Small pseudoaneurysms - May attempt until invasive methods are available	- Radial artery occlusion - Venous occlusion - Hand swelling
Thrombin injection	- Minimally invasive - Short recovery time	- Requires expertise	- Neck length >3 mm - Arteriotomy diameter <5 mm	- Embolization - Allergic reaction to recombinant thrombin
Percutaneous - Covered stents - Endovascular coils - Long sheaths	- Minimally invasive - Short recovery time	- Requires expertise - Radiation exposure - Contrast use	- Suitable peripheral anatomy for access and intervention	- Coil embolization - Bleeding complications - Acute kidney injury - Radial artery occlusion
Surgery	- Definitive treatment	- Invasive - Long recovery - High risk in older patients with multiple comorbidities	- Compartment syndrome, neurological symptoms or other conditions requiring urgent and definitive intervention - Concomitant complications that benefit from surgical exploration (eg, fistula)	- Postsurgical infections - Injury to surrounding structures - Scarring - Anesthesia complications

A summary of the strengths and limitations of each method, in addition to a guide on when to use each method.

US, ultrasound.

### Prevention

Preventing radial artery pseudoaneurysms revolves around controlling risk factors, adopting proper radial artery access techniques, and performing proper postangiography hemostasis and monitoring.

Risk factor control includes cessation of DOAC use for at least 24 hours prior to the procedure and checking INR in patients taking warfarin before intervention. Discontinuing DOAC for longer periods may be needed in patients with a higher risk of bleeding or kidney dysfunction. Coronary angiography should be performed with the smallest sheath diameter possible and using ultrasonography guidance to possibly limit the number of arteriotomies performed and statistically decrease the chance of developing vascular complications.

Postangiography care is essential in prevention and includes applying pressure at the access site in all cases. Using a compression device (inflatable cuff) is the most common method of achieving hemostasis, and when used, patent hemostasis should be achieved. This ensures enough pressure application to prevent bleeding without being high enough to cause occlusion. We propose a similar method in applying pressure in treating radial artery pseudoaneurysm, where the inflation pressure should be high enough to stop the flow to and from the pseudoaneurysm without being high enough to cause occlusion.

Close postangiography monitoring by assessing the access site daily in inpatient cases and within the first week postangiography in outpatient cases is essential in the early detection and prevention of further propagation. It is important as well to educate patients on the possible complications after radial access and the different signs of symptoms of the common access complications, and encourage them to seek medical attention in case of occurrence.

### Limitations

The fact that most of the publications included in this systematic review are narrative reports of individual patients leaves them prone to different types of biases. This includes a publication bias, where cases with successful treatment are published, whereas others that failed are not. In addition, there may be a selection bias as the decision to select a specific method over another may have been made based on the lack of availability of certain methods rather than on the suitability of the case for that specific method, which reflects the available expertise in the specific hospitals that generated the cases. Lastly, most of the included publications discuss the acute management of this complication without reporting any long-term outcomes or recurrence rates, if any. Such long-term data are important and may change the decision to select certain methods.

It is important to note as well that there is great variation in which data were reported between the different publications. This resulted in a big variation between the sample sizes of each studied effect. For example, a successful management method was reported in all 75 patients, whereas anticoagulant use was reported in only 60 patients. Sample sizes for each reported data category are included in the results table (Table 1).

Despite these limitations, we believe that this paper provides general guidance to providers on dealing with this complication and provides an outline on using the available expertise in management. It also brings attention to this rare complication and encourages operators to publish radial artery pseudoaneurysm cases, the selected management strategy, and the outcomes. Building on the methodology and results of this paper, we encourage exploring large databases and prospective registries that document this complication, which may provide a larger sample size, more detailed risk factors, and more information regarding management strategies.

## Conclusion

Radial artery pseudoaneurysm is a rare complication of transradial coronary angiography. Although a relationship between chronic anticoagulant use and developing radial artery pseudoaneurysm could not be proven, there was a higher proportion of warfarin use in patients who developed this complication compared to enoxaparin or DOAC.

Compression is a documented noninvasive management method. Despite its common use, it has the lowest efficacy, with a 41.5% failure rate necessitating invasive treatment. Thrombin injection should be preceded by ultrasonographic measurements of the lesion to predict suitability, which includes neck length and arteriotomy diameter. Percutaneous interventions and surgery are other invasive methods that are curative.

Multidisciplinary discussion should guide the best management strategy, taking into account patient preferences, pseudoaneurysm dimensions, and the available expertise.
