# Supplementary material for: Managing Postangiography Radial Artery Pseudoaneurysms: Systematic Review of the Published Literature
Source: J Soc Cardiovasc Angiogr Interv. 2026 Feb 24;5(3):104263. doi: 10.1016/j.jscai.2026.104263 (PMC13005397; doi:10.1016/j.jscai.2026.104263)
Supplement: Supplementary Table 1 [file mmc1.docx]

|  | Database | Study Type | First author | | Year | | Country | | Patient | | Age | | Sex |
| --- | --- | --- | --- | --- | --- | --- | --- | --- | --- | --- | --- | --- | --- |
| 1 | Pubmed | Case report | | Gupta A | | 2021 | | India | | 1 | | 53 | M |
| 2 | Pubmed | Case report | | Cunha R | | 2023 | | Portugal | | 2 | | 62 | F |
| 3 | Pubmed | Case report | | Kumar V | | 2016 | | India | | 3 | | 54 | F |
| 4 | Pubmed | Case report | | Swanton A | | 2019 | | Australia | | 4 | | 85 | F |
| 5 | Pubmed | Case report | | Emrich I | | 2023 | | Germany | | 5 | | 80 | F |
| 6 | Pubmed | Case report | | Prakash B | | 2021 | | India | | 6 | | 82 | M |
| 7 | Pubmed | Case report | | Sinha S | | 2021 | | India | | 7 | | 68 | F |
| 8 | Pubmed | Case report | | Sharma R | | 2021 | | USA | | 8 | | 77 | F |
| 9 | Pubmed | Case report | | Berrio-Caicedo J | | 2022 | | Colombia | | 9 | | 74 | M |
| 10 | Pubmed | Case report | | Korabathina R | | 2015 | | USA | | 10 | | 56 | M |
| 11 | Pubmed | Case report | | Papadoulas S | | 2022 | | Greece | | 11 | | 83 | M |
| 12 | Pubmed | Case report | | Tsiafoutis I | | 2020 | | Greece | | 12 | | 82 | M |
| 13 | Pubmed | Case report | | Samaranayake C | | 2015 | | New Zealand | | 13 | | 85 | F |
| 14 | Pubmed | Case report | | Maznyczka A | | 2018 | | United Kingdom | | 14 | | 93 | F |
| 15 | Pubmed | Case report | | Petersen J | | 2015 | | Spain | | 15 | | 81 | F |
| 16 | Pubmed | Case report | | Oliveira M | | 2023 | | Brasil | | 16 | | 50 | M |
| 17 | Pubmed | Case report | | Pacha H | | 2018 | | USA | | 17 | | 67 | F |
| 18 | Pubmed | Retrospective study | | Lotan C | | 1995 | | Israel | | 18 | | ---- | ---- |
| 18 | Pubmed | Retrospective study | | Lotan C | | 1995 | | Israel | | 19 | | ---- | ---- |
| 19 | Pubmed | Case report | | Alqahtani N | | 2024 | | Saudi Arabia | | 20 | | 83 | M |
| 20 | Pubmed | Case report | | Ghanavati R | | 2017 | | Iran | | 21 | | 32 | M |
| 21 | Pubmed | Case report | | Rafeh N | | 2012 | | USA | | 22 | | 73 | F |
| 22 | Pubmed | Case report | | Mahanta D | | 2020 | | India | | 23 | | 74 | M |
| 23 | Pubmed | Case series | | Hamid T | | 2012 | | United Kingdom | | 24 | | 83 | F |
| 23 | Pubmed | Case series | | Hamid T | | 2012 | | United Kingdom | | 25 | | 80 | F |
| 24 | Pubmed | Case report | | Babunashvili A | | 2017 | | Russia | | 26 | | 68 | M |
| 25 | Pubmed | Case report | | Kiat J | | 2020 | | Singapore | | 27 | | 76 | F |
| 26 | Pubmed | Case report | | Herold J | | 2011 | | Germany | | 28 | | ---- | ---- |
| 27 | Pubmed | Case report | | Bauer P | | 2014 | | Germany | | 29 | | 65 | M |
| 28 | Pubmed | Case report | | Wu L | | 2019 | | USA | | 30 | | 75 | F |
| 29 | Pubmed | Case report | | Tsiafoutis I | | 2018 | | Greece | | 31 | | 65 | M |
| 30 | Pubmed | Case series | | Palaparti R | | 2019 | | India | | 32 | | 65 | M |
| 30 | Pubmed | Case series | | Palaparti R | | 2019 | | India | | 33 | | 74 | F |
| 30 | Pubmed | Case series | | Palaparti R | | 2019 | | India | | 34 | | 69 | F |
| 30 | Pubmed | Case series | | Palaparti R | | 2019 | | India | | 35 | | 69 | F |
| 31 | Pubmed | Case report | | Williams P | | 2009 | | United Kingdom | | 36 | | 79 | F |
| 32 | Pubmed | Case report | | Mohamed M | | 2015 | | United Kingdom | | 37 | | 85 | F |
| 33 | Pubmed | Case series | | Janus B | | 2016 | | Poland | | 38 | | 76 | F |
| 33 | Pubmed | Case series | | Janus B | | 2016 | | Poland | | 39 | | 86 | F |
| 34 | Pubmed | Case report | | Suchon E | | 2013 | | Poland | | 40 | | 85 | F |
| 35 | Pubmed | Case report | | Spence M | | 2009 | | Canada | | 41 | | 59 | M |
| 36 | Pubmed | Case report | | Inan M | | 2011 | | Turkey | | 42 | | 65 | M |
| 37 | Pubmed | Case report | | Colletti G | | 2024 | | Belgium | | 43 | | 82 | M |
| 38 | Pubmed | Case series | | Collins N | | 2012 | | Australia | | 44 | | 82 | F |
| 38 | Pubmed | Case series | | Collins N | | 2012 | | Australia | | 45 | | 58 | F |
| 38 | Pubmed | Case series | | Collins N | | 2012 | | Australia | | 46 | | 44 | M |
| 38 | Pubmed | Case series | | Collins N | | 2012 | | Australia | | 47 | | 61 | F |
| 38 | Pubmed | Case series | | Collins N | | 2012 | | Australia | | 48 | | 66 | F |
| 39 | Pubmed | Case report | | Cauchi M | | 2014 | | USA | | 49 | | 45 | M |
| 40 | Pubmed | Case report | | Gallinoro E | | 2019 | | Italy | | 50 | | 84 | F |
| 41 | Pubmed | Case report | | Iftikhar S | | 2019 | | USA | | 51 | | 82 | F |
| 42 | Pubmed | Case report | | Alerhand S | | 2018 | | USA | | 52 | | 57 | F |
| 43 | Pubmed | Case report | | Blasco A | | 2005 | | Spain | | 53 | | 55 | M |
| 44 | Pubmed | Case report | | Bhat T | | 2013 | | USA | | 54 | | 80 | F |
| 45 | Pubmed | Case report | | Molina-Lopez V | | 2021 | | USA | | 55 | | 82 | M |
| 46 | Pubmed | Case report | | Nykl R | | 2021 | | Czech | | 56 | | 60 | F |
| 47 | Pubmed | Case series | | Zegri I | | 2015 | | Spain | | 57 | | 55 | M |
| 47 | Pubmed | Case series | | Zegri I | | 2015 | | Spain | | 58 | | 76 | M |
| 47 | Pubmed | Case series | | Zegri I | | 2015 | | Spain | | 59 | | 79 | M |
| 47 | Pubmed | Case series | | Zegri I | | 2015 | | Spain | | 60 | | 88 | M |
| 47 | Pubmed | Case series | | Zegri I | | 2015 | | Spain | | 61 | | 81 | F |
| 48 | Scopus | Case report | | Ponna P | | 2023 | | USA | | 62 | | 73 | M |
| 49 | Scopus | Case report | | Campbell S | | 2022 | | New Zealand | | 63 | | 74 | M |
| 50 | Scopus | Case report | | Pinxterhuis T | | 2022 | | The Netherlands | | 64 | | 71 | M |
| 51 | Scopus | Case report | | Li Y | | 2021 | | China | | 65 | | 66 | F |
| 52 | Scopus | Case report | | Stecko W | | 2020 | | Poland | | 66 | | 82 | F |
| 53 | Scopus | Case report | | Sinha S | | 2017 | | India | | 67 | | 43 | F |
| 54 | Google scholar | Case series | | Tosti R | | 2017 | | USA | | 68 | | 83 | F |
| 54 | Google scholar | Case series | | Tosti R | | 2017 | | USA | | 69 | | 72 | F |
| 54 | Google scholar | Case series | | Tosti R | | 2017 | | USA | | 70 | | 56 | F |
| 54 | Google scholar | Case series | | Tosti R | | 2017 | | USA | | 71 | | 61 | M |
| 54 | Google scholar | Case series | | Tosti R | | 2017 | | USA | | 72 | | 60 | M |
| 54 | Google scholar | Case series | | Tosti R | | 2017 | | USA | | 73 | | 77 | F |
| 55 | Google scholar | Case report | | Kis M | | 2021 | | Turkey | | 74 | | 78 | F |
| 56 | Google Scholar | Case report | | Baris V | | 2016 | | Turkey | | 75 | | 73 | F |

**Supplemental Table S1: Studies included in the final analysis.** Final analysis included 56 studies describing 75 patients from 24 countries. Age and sex of patients are included. M: male, F: female.
